# Prescribing Immunoglobulin Replacement Therapy for Patients with Non-classical and Secondary Antibody Deficiency: an Analysis of the Practice of Clinical Immunologists in the UK and Republic of Ireland

**DOI:** 10.1007/s10875-017-0469-4

**Published:** 2018-02-08

**Authors:** John David M. Edgar, Alex G. Richter, Aarnoud P. Huissoon, Dinakantha S. Kumararatne, Helen E. Baxendale, Claire A. Bethune, Tomaz Garcez, Siraj A. Misbah, Ricardo U. Sorensen, United Kingdom Primary Immunodeficiency Network (UKPIN) Immunoglobulin Decision to Treat Study Group

**Affiliations:** 1 Regional Immunology Service, The Royal Hospitals, Belfast Health & Social Care Trust and Queen’s University Belfast, Belfast, Northern Ireland UK; 2 Clinical Immunology Service, Birmingham Medical School & University Hospitals, Birmingham NHS Foundation Trust, Queen Elizabeth Hospital, Birmingham, B15 2GW UK; 3 West Midlands Immunodeficiency Centre, Birmingham Heartlands Hospital, Birmingham, UK; 4 Department of Clinical Biochemistry and Immunology, Box 109, Addenbrooke’s Hospital, Cambridge, CB2 0QQ UK; 5 Papworth Hospital NHS Foundation Trust, Papworth Everard, Cambridge, CB23 3RE UK; 6 Department of Clinical Immunology, Plymouth Hospitals NHS Trust, Plymouth, UK; 7 Manchester University NHS Foundation Trust, Manchester, UK; 8 Department of Clinical Immunology, Oxford University NHS Trust, Oxford, OX3 9 DU UK; 9 Clinical Professor of Pediatrics, Department of Pediatrics, Louisiana State University Health Science Center, New Orleans, Louisiana, USA & Honorary Professor, Faculty of Medicine, University of La Frontera, Temuco, Chile

**Keywords:** Immunoglobulin replacement, immunodeficiency, diagnosis, therapy, vaccine responses

## Abstract

**Background:**

Immunologists are increasingly being asked to assess patients with non-classical and secondary antibody deficiency to determine their potential need for immunoglobulin replacement therapy (IGRT). Immunoglobulin is a limited, expensive resource and no clear guidance exists for this broad patient group. The purpose of this survey is to establish what factors influence the decision to commence IGRT in adult patients, when diagnostic criteria for primary antibody deficiency are not fulfilled.

**Methods:**

Under the auspices of the United Kingdom Primary Immunodeficiency Network (UKPIN), a study group was established which circulated an online questionnaire to the consultant body across the UK and Ireland. Results provided a snapshot of the current clinical practice of 71% of consultant immunologists, from 30 centers.

**Results:**

In order of importance, factors which influence the decision to commence IGRT include number of hospital admissions with infection, serum IgG level, bronchiectasis, radiologically proven pneumonia, number of positive sputum cultures, number of antibiotic courses, and results of immunization studies. The commonest test vaccine used was Pneumovax 23 with measurement of serotype-specific responses at 4 weeks, with a threshold of 0.35 μg/ml in 2/3 of serotypes measured. Eighty-six percent of patients are treated with a trial of prophylactic antibiotics prior to consideration of IGRT. Efficacy of IGRT trial is assessed at between 6 and 12 months.

**Conclusions:**

There was consistency in clinical practice using a combination of clinical history, evidence of infections, and vaccination testing for diagnosis. However, there was some variation in the implementation of this practice, particularly in vaccine choice and assessment of response to vaccination.

## Background

Immunoglobulin replacement therapy (IGRT) is a mainstay in the management of patients with antibody deficiency [1, 2]. IGRT is delivered either by the intravenous (IVIG) or subcutaneous (SCIG) routes either in hospital or at home. Antibody deficiency can be primary (PID) or secondary (SID) to an underlying disorder, such as chronic lymphocytic leukaemia or immunosuppressive treatment. The diagnosis of antibody deficiency can be clear-cut, as in X linked agammaglobulinemia, where there is a complete absence of antibodies and B cells. However, more often, the diagnosis is less explicit and immunologists use a combination of clinical history and laboratory investigation including test vaccination and serological testing to make a diagnosis [3]. The absence of a single laboratory or clinical parameter to predict response to IGRT is challenging, and in practice, this is usually assessed using a combination of clinical response and serum IgG level.

Therapeutic immunoglobulin is a limited, relatively expensive resource, and in the past, there have been intermittent interruptions of supply because of occasional manufacturing difficulty or quality control failures. It is therefore imperative that immunoglobulin is used carefully, and to this end in 2008, the Department of Health (DoH, England and Wales) introduced clinical guidance on the use of IGRT, which covers approved indications, recommended dosing, and monitoring. This guidance, updated in 2012, (https://www.gov.uk/government/publications/clinical-guidelines-for-immunoglobulin-use-second-edition-update) includes a colour-coded classification to prioritise immunoglobulin prescription across all specialities for a wide range of conditions including IGRT and immunomodulatory usage. These guidelines are based on the strength of evidence, expert opinion, and the availability of alternative therapies for these medical conditions. Based on this classification, DoH approval for the use of IGRT is automatically granted (red), approved when supply is not compromised (blue), granted if alternate therapy unavailable or ineffective (grey), and not normally granted (black). Hospitals and regions have established immunoglobulin advisory panels (IAP) which include representatives of relevant clinical specialities, pharmacy, and commissioner stakeholders, to ensure that the guidance is fully implemented. A national immunoglobulin registry is also established to collect data on usage nationally.

The purpose of this survey is to establish what factors most influence colleagues to commence IGRT in adult patients, when strict diagnostic criteria for CVID, XLA, and other well-defined PIDs are not fulfilled. Definition of this patient group is challenging, but would include patients with a > 2SD reduction in a single class of immunoglobulin (IgG/IgA or IgM), IgA or IgG subclass deficiency accompanied by impaired responses to immunization and a history of recurrent sinopulmonary infection. In the DoH guidance, primary antibody deficiency (including specific antibody deficiency) is classified as a red indication. Criteria for the diagnosis of specific antibody deficiency (SPAD) are not defined but the guidelines state the diagnosis must be established by a clinical immunologist. SID due to any cause is recognized as a blue indication. The SID diagnostic criteria are more specific and require a history of infections in the presence of hypogammaglobulinaemia, failure to respond to polysaccharide vaccine challenge, and a lack of response to a 3-month course of prophylactic antibiotics.

In general, lower serum IgG concentrations are associated with an increased risk of infection. However, serum IgG alone is not a definitive biomarker. Patients may have very low IgG levels but not suffer from infections, or patients with normal IgG levels may demonstrate susceptibility to severe or recurrent infection and may be diagnosed with specific antibody deficiency (SPAD). SPAD is characterised as a failure to respond normally to test immunization with a polysaccharide vaccine in the context of a normal total level of IgG [4]. There is however, no consensus as to what constitutes a “normal” response to polysaccharide immunization. In addition, normal range responses in patients with co-morbidities has not been well established [5]. It is also widely recognised that inter-assay variability may be considerable such that individual laboratories should establish “normal” range responses for polysaccharide antibody assays [6, 7]. Despite this uncertainty, in the UK, SPAD qualifies as a red indication under the DoH approval guidelines, which effectively allows automatic approval of IGRT.

The detail of what constitutes a significant infection history, what defines a low IgG level, which vaccine or prophylactic antibiotic to use, and when and how to measure the response to vaccination or treatment is not prescribed, and so the diagnosis of SPAD remains at the discretion of a clinical immunologist. This lack of diagnostic certainty leads to differing opinions and there is likely to be some variation in practice across different centers. To address this, a UKPIN audit steering group was assembled to undertake a survey of UK and Irish consultant immunologists to establish current practice in relation to the decision to commence IGRT in their practice.

## Methods

The steering group was formed from the immunology consultant body. As this project was an audit of practice, rather than research, ethical approval was not required. An online questionnaire was developed on the Survey Monkey platform (SurveyMonkey Inc., San Mateo, CA, USA www.surveymonkey.com: appendix 1) and following approval from the steering group, it was circulated by email to the UK and Ireland Immunology consultant body via the UK Primary Immunodeficiency Network (UKPIN) circulation list, and a second consultant membership list called Travellers. In total, 59 potentially eligible consultants were identified.

### Presentation of Data

Results are largely reported as descriptive statistics and no formal statistical comparisons or analyses are made. In some questions, respondents were asked to rank certain factors in order of priority and this generated “ranking scores” for each answer choice. The answer choice with the highest average ranking is the most preferred choice. The average ranking was calculated by the Survey Monkey platform as follows: *w* = weight of ranked position, *y* = response count for answer choice. Rank score = *y*1*w*1 + *y*2*w*2 + *y*3*w*3... *ynwn*. Weights were applied in reverse. In other words, the respondent’s most preferred choice (which they rank as #1) had the highest weight and their least preferred choice (which they ranked in the last position) had a weight of 1. For example, if a ranking question has five answer choices, weights were assigned as follows: the #1 choice had a weight of 5, the #2 choice had a weight of 4, the #3 choice had a weight of 3, the #4 choice had a weight of 2, and the #5 choice had a weight of 1. Any N/A responses did not factor into the average ranking.

## Results

Fifty-seven individuals opened the email and 42 consultant immunologists from the UK and Republic of Ireland responded to the survey. This represents 71% of practicing consultant immunologists in the UK and Ireland with a cumulative total of 480 years of consultant experience (mean 11.42 years). Data were collected from 30 separate centers in three of the four countries of the UK (England (*n* = 33), Scotland (*n* = 3), Northern Ireland (*n* = 3), and the Republic of Ireland (*n* = 2). One response was anonymous. The survey reveals that UK immunologists are regularly being asked to consider IGRT in patients referred to the outpatient clinic (Table 1) with primary and secondary antibody deficiency. 29.3% of immunologists undertake test immunization in all patients and the remaining 70.7% undertake this in the majority of cases.

**Table 1:** ᅟSurvey of UK and Irish Immunologists in relation to referral and assessment of patients with suspected nonclassical or secondary immunodeficiency

Question 1	Answers	*N* (%)
1. How often do you see patients as described above, in whom you are asked to consider immunoglobulin replacement therapy (IGRT)?	Almost every clinic	25 (59.5)
	Quite often	16 (38.1)
	Occasionally	1 (2.4)
	Rarely	0 (0)
2. Do you see referrals from other specialties (e.g. haematology, rheumatology) with suspected secondary antibody deficiency related to disease or drug therapy?	Almost every clinic	18 (42.86)
	Quite often	20 (47.62)
	Occasionally	3 (7.1)
	Rarely	1 (2.4)
3. In assessing immune function, prior to commencing IGRT do you undertake test immunisation?	In all patients	12 (29.3)
	In the majority of patients	29 (70.7)
4. If undertaking test immunisation, which of the following vaccines do you regularly use?	Pneumococcal Polysaccharide Vaccine, 23-Valent (PPV23)	40 (95.2)
	Meningitis ACWY conjugated	2 (4.8)
	Prevenar 13	24 (57.1)
	Tetanus toxoid (combined vaccine)	31 (73.8)
	Haemophilus Influenza B (HiB)	30 (71.4)
	Salmonella Typhi Vi	4 (9.5)

### Pneumococcal Vaccination

Pneumococcal vaccination was anticipated to be the most widely used vaccine so further questions were asked to elucidate UK practice (Table 2). Pneumococcal polysaccharide vaccine, 23-valent (PPV23) was found to be the commonest vaccine used to examine immune responses (95.2%) although HiB (used by 71.4%), tetanus (73.8%) and Prevenar13 (73.8%) were frequently used.

**Table 2:** ᅟSurvey to elucidate UK practice about pneumococcal vaccination

Question	Answer options	*N* (%)
5. If you use both Prevenar 13 and Pneumococcal Polysaccharide Vaccine, 23-Valent (PPV23). In which order do you administer these?	• PPV23 first then Prevenar 13	26 (68.4)
	• Prevenar 13 first then PPV23	3 (7.9)
	• No fixed order	0 (0)
	• Administer both simultaneously	0 (0)
	• Other	9 (23.7)
6. How long after immunisation do you recommend “post vaccination samples” be taken?	• 4 weeks	42 (100)
7. In assessment of pneumococcal antibody responses, which methodology do you use?	• Total IgG anti-pneumococcal antibody	22 (52.4)
	• Total IgG2 anti pneumococcal antibody	4 (9.5)
	• pneumococcal serotype specific antibody (PSSA) (ELISA)	8 (19.0)
	• PSSA (bead based assay)	32 (76.2)
8. In interpretation of PSSA results, which “cut-off” levels do you use?	• 0.35 mcg/ml	31(79.5)
	• 0.5 mcg/ml	0 (0)
	• 1.3 mcg/ml	0 (0)
	• A combination of the above	8 (20.5)
9. When interpreting PSSA results, what percentage of serotypes do you expect to exceed your chosen “cut-off” in a normal response (choose the % threshold closest to your current practice)	For 0.35 mcg/ml (35 responders)	
	30% of serotypes protected	0 (0.0)
	50% of serotypes protected	8 (22.8)
	66% of serotypes protected	24 (68.6)
	90% of serotypes protected	3 (8.6)
	For 1.3 mcg/ml (8 responders)	
	30% of serotypes protected	3 (37.5)
	50% of serotypes protected	1 (12.5)
	66% of serotypes protected	3 (37.5)
	90% of serotypes protected	1 (12.5)
10. How do you regard the value of pneumococcal antibody response testing in the decision to commence IGRT?	• I regard this as a definitive diagnostic test	0 (0.0)
	• I rely on this in the majority of cases	6 (14.6)
	• I rely on this to support my clinical assessment	28 (68.3)
	• I rely on this, unless it appears to conflict with the clinical history	4 (9.8)
	• I am sceptical of the value and disregard this in some cases	3 (7.3)
	• I quite often disregard this	0 (0)

Test responses were unanimously examined at 4 weeks (with some flexibility noted from free text comments). Vaccine responses were most commonly measured by pneumococcal serotype-specific antibody (PSSA) responses by bead-based assays or ELISA, but the survey numbers suggest that many immunologists use both this and a whole pneumococcal assay. When PSSA is used, most use a cut-off concentration of 0.35 μg/ml. An adequate response is considered by the majority of users to be achieved when > 66% of serotypes are in the protective range, regardless of the cut-off concentration. Immunologists predominantly use pneumococcal antibody testing to assess B cell function as a complementary tool to support clinical assessment but would not regard this as a definitive diagnostic test.

### Decision Criteria to Commence IGRT

The survey aimed to understand the factors that influenced the decision to commence IGRT. These factors were then ranked by the responders from most to least important (Question 11: Table 3).

**Table 3:** ᅟRanking of factors that influenced the decision to commence IGRT

Q.11. How do you rank the following factors in influencing your decision to commence IGRT?	
Criteria	Rank score
Number of hospital admissions with infection	11.36
Total serum IgG level	10.38
Presence of bronchiectasis	10.07
Radiologically proven pneumonia	9.90
Number of positive sputum cultures	9.02
Number of courses of antibiotics (GP or pharmacy confirmed)	8.72
Results of Immunisation studies	8.33
Types of bacteria isolated in sputum	8.05
Failure to improve on antibiotic prophylaxis	7.38
Number of self-reported chest/sinus/ear infections	6.99
Number of courses of antibiotics (patient reported)	6.64
Other structural lung disease	4.32
Self-reported “well-being”	2.95
Formal quality of life assessment (including validated questionnaires, visual analogue scales etc.)	2.87

The results show that no single criterion is used but a combination of significant infection; low immunoglobulin and evidence of end organ damage are considered by responders the most important.

Given the importance of clinical assessment in commencing patients on IGRT, radiological practice was also considered. Responses to question 12 indicated that high-resolution CT scanning is routinely undertaken at initial assessment by 34 (87.2%) of respondents (all cases by *n* = 11 (28.2%); majority of cases by *n* = 23 (59.0%)). HRCT scanning was restricted to those cases where a daily productive cough suggests the presence of bronchiectasis by *n* = 4 (10.3%) and only rarely by *n* = 1 (2.6%).

### Use of Prophylactic Antibiotics (Questions 13 and 14)

Prophylactic antibiotic prescribing prior to commencing IGRT was also commonly implemented: prescribed in all patients by *n* = 9 (21.4%) immunologists, in the majority of patients by *n* = 27 (64.3%), in a minority of patients by *n* = 5 (11.9%), rarely *n* = 1 (2.4%), and never by *n* = 0 (0.0%).

The first choice of antibiotic varied between consultants with the commonest being azithromycin followed by doxycycline and amoxicillin (Table 4).

**Table 4:** ᅟRanking of antibiotics as suitable for prophylaxis in a patient group

Q 14. How would you rank the following antibiotics as suitable for prophylaxis in this patient group?	
Antibiotic	Ranking score
Azithromycin	7.36
Doxycycline	6.12
Amoxicillin	6.05
Co-trimoxazole	5.11
Co-amoxiclav	4.42
Ciprofloxacin	3.18
Cefalexin	2.88
Penicillin	2.80

Importantly, responses to question 15 (not shown: If a patient fails to improve on a trial of prophylactic antibiotics, do you consider a trial of IGRT?) indicated that all immunologists would consider a trial of IGRT in at a least some patients who fail to improve on a trial of prophylactic antibiotics: responses: in all patients *n* = 2 (4.8%), in the majority of patients *n* = 34 (81.0%), and in a minority of patients *n* = 6 (14.3%).

The responses to question 16 (not shown: What initial dose of IGRT do you use?) indicate a dichotomy of approach in terms of immunoglobulin dosing. Twenty-two over forty-two (52%) respondents commence all patients on 400 mg/kg/month. In 19/42 (45%) however, the presence of bronchiectasis dictates a higher starting dose of 600 mg/kg/month. One respondent (2.5%) used a dose of 200 mg/kg/month and 400 mg/kg/month for patients in the same situations. Two respondents also commented they would use a higher starting dose if the patient had bad gastrointestinal or other autoimmune disease.

Responses to question 17 (not shown: After what time interval would you usually assess response to immunoglobulin replacement therapy to determine whether it should be continued?) indicated that immunologists use a therapeutic trial period to assess response to immunoglobulin replacement therapy before committing to lifelong therapy. The minimum interval used was 6 months (*n* = 13, 31.7%), two respondents (4.9%) used a 9 month window, whereas the most common time period was 12 months with 26 (63.4%) favouring this choice.

The factors that most influenced immunologists to consider the trial to have been successful were reduction in frequency of reported infection, hospital admission, and antibiotic prescriptions (Table 5).

**Table 5:** ᅟThe factors that most influenced immunologists to consider the trial to have been successful

Factor	Ranking score
Reduction in frequency of reported infections	5.98
Reduction in hospital admissions	5.93
Reduction in antibiotic prescription frequency	5.65
Trough/random IgG levels	3.72
Improvement in self-reported well-being	3.31
Formal quality of life assessment	3.00

We found no identifiable regional differences in responses by location (England, Scotland, Northern Ireland, and Republic of Ireland). Nor did free text comments indicate any region/nation specific differences in practice.

## Discussion

Primary antibody deficiencies have a prevalence of 2.11/100,000 in the UK [8] The increased survival from cancer and use of immunosuppressive agents in auto-inflammatory conditions and transplantation is causing an increasing, but poorly quantified burden of secondary immunodeficiency [9, 10].

The results of this survey represent the practice of the majority of consultant immunologists currently practicing in the UK and Ireland (71%), with a cumulative total of 435 years of consultant experience. This is to our knowledge the first supra national survey of current clinical practice in this area. This survey confirms that UK clinical immunologists are frequently asked to evaluate patients, with suspected antibody deficiency, to consider commencement of IGRT. Results show that there is consistency in using a combination of clinical history, evidence of infections, and vaccination testing for diagnosis. However, there is variation in the implementation of this practice, particularly in vaccine choice and assessment of response to vaccination.

All respondents always or usually undertook immunization studies and recommended a 4-week interval between pre- and post-immunization samples. As expected, polysaccharide pneumococcal vaccination was most commonly used, as SPAD is defined as a failure to respond to polysaccharide vaccination. An additional conjugate pneumococcal vaccine is advised by many immunologists to provide enhanced therapeutic benefit.

There is some variation in how vaccine responses are assessed including which assay to use or whether to measure combined or individual pneumococcal serotype responses. Whole pneumococcal assays usually assess the total IgG reactive against 23 serotypes present in the pneumococcal polysaccharide vaccine, 23-valent (PPV23) vaccine. Less than 10% of immunologists use IgG2 subclass assays. The advantage of the whole pneumococcal assay is that the test is relatively inexpensive and there is a single value output. However, these tests do not identify abnormal responses to individual serotypes and may therefore be falsely reassuring [11]. Furthermore, there is no correlation in the results obtained by whole pneumococcal ELISA, and measuring antibody levels to individual pneumococcal serotypes [11]. In contrast, bead-based or ELISA methods that assess serotype-specific IgG concentrations do provide this detail. However, this additional data poses challenges in interpretation both in defining the percentage of serotypes that would be expected to elicit an antibody response and the threshold level for protection against invasive and non-invasive pneumococcal disease (IPD, n-IPD, respectively) [12].

An IPD protective level of 0.35 μg/ml was proposed by the WHO following collation of three large conjugate vaccine studies in neonates. A much higher nIPD protective level of 1.3 μg/ml was proposed following analysis of US data using PPV23. A further consideration is that it is likely that not all serotype-specific antibodies are equally protective and therefore simply counting the percentage response may be misleading. It can further be argued that defining a protective level is irrelevant to the diagnosis of immunodeficiency. The aim of vaccination studies here is simply to differentiate an abnormal from a normal immune response. In the absence of definitive evidence, this survey reveals that the majority opinion in the UK is to use 0.35 μg/ml in two thirds of serotypes measured as an indicator of a “normal response.” The inconsistencies in antibody assessment methods may also explain why evaluation of vaccine responses are not considered a definitive test.

It is also noteworthy that a recent study of antibody responses in patients with chronic sinusitis indicated that 11% of healthy controls would fulfil diagnostic criteria for SPAD, and this lack of certainty in the definition of SPAD is likely to contribute to variation in practice [13].

Prophylactic antibiotics are used by 85% UK immunologists to reduce infection rates. The choice of antibiotic naturally depends on bacterial sensitivities (where available) and also intolerance and allergy. Prophylactic antibiotic usage can be controversial in an era of increasing antibiotic stewardship. However, immunoglobulin is a scarce and expensive resource and alternative strategies should be considered.

The ranking of factors influencing the commencement of immunoglobulin provided some of the most interesting data. Despite intense interest in the assessment of immunization responses as an indicator of immune function [2, 12], these were ranked only 7th out of 14 factors that most influenced the decision to commence IGRT. Conventional direct and indirect clinical indicators of recurrent chest infection all ranked higher in the decision-making hierarchy, with a bias toward the more objective (number of proven infections, pharmacy confirmed prescriptions, etc) compared to self-reported illness. An important finding is that self-reported “well-being” and formal quality of life assessments were ranked as the least important of all factors considered. Whilst improved quality of life and well-being are clearly worthwhile clinical goals, they are too non-specific and multifactorial to be used as indicators of the need for (or response to) immunoglobulin therapy.

There was consistency in the use of baseline HRCT chest in the assessment of patients and the majority were in favour of a 12-month trial period for IGRT (63.4% of respondents). The identification that approximately half of colleagues use an initial starting dose of 400 mg/kg/month for all patients whilst approximately the other 50% use a higher starting dose for those with perceived complications/end organ damage is in line with recent recommendations ([2, 14]. Free text responses from many indicated that treatment is tailored to the needs of the individual patient. A specific question about any differences in dosage of IgG used to treat secondary antibody deficient versus specific antibody deficiency was not included in this survey; however, free text comments did not indicate any difference in practice. It therefore appears that common practice by the immunologists in the UK and Ireland is to treat all patients considered to have an antibody deficiency with similar IgG doses. Initial dose selection is adjusted for complications like the presence of bronchiectasis by 45% of respondents.

The ranking of factors used to judge success of a trial of IGRT was not surprisingly focused on reduction in number of infections, hospital admissions, and numbers of prescribed antibiotic courses. This survey did not explore the importance of intravenous (IV) versus oral antibiotics prior usage in the decision-making. A previous paediatric study found that prior usage of IV antibiotics was a predictor of neutrophil PID in particular [15], and this may be an issue worthy of future investigation in non-classical or SID.

This survey provides a valuable snapshot of the current practice of immunologists in the UK and Ireland in how they approach the decision to treat adults with non-classical PID or suspected SID with IGRT. SID has clear diagnostic criteria in terms of a serum IgG < 5 g/L accompanied by failure to respond to polysaccharide vaccine challenge. In PID, in addition to immunoglobulin deficiencies, one of the accepted criteria is a specific antibody deficiency. However, the diagnosis of SPAD is based on the sole immunological criterion of failure to respond to polysaccharide vaccine challenge that can be identified using a variety of methodologies and interpretation choices. The vaccines used to assess B cell function coupled with differing definitions of what constitutes a “normal” response to test immunization with pneumococcal vaccines suggests that eligibility criteria for IGRT in SPAD are likely to be subject to substantial variation and attract controversy [14].

The outcomes demonstrate that the decision-making process is multifactorial and complex, and some factors are not mutually exclusive. There is no single universally accepted biomarker to predict clinically significant antibody deficiency; in addition to laboratory test results, most UK and Irish immunologists rely heavily on the clinical presentation of a patient to identify those who may require long term IGRT.

## Take Home Messages

- Immunologists frequently see patients who do not fulfil classical definitions of PID for the assessment of suitability for IGRT
- Test immunisation with pneumococcal vaccines is widely used however there is variability on assessing a “ normal “ response
- Whilst all clinicians use test immunisation in their assessment, most do not regard this as a definitive diagnostic test . Objective clinical indicators of frequency and severity of infection tend to take precedence over immunisation studies in determining the need for IGRT
- Prophylactic antibiotics are commonly used as a precursor to IGRT
- Immunologists in the UK and Ireland commonly use a “trial of IGRT” usually for 12 months ( 63.4% of respondents) to assess its clinical efficacy in individual patients

**United Kingdom Primary Immunodeficiency Network (UKPIN) Immunoglobulin Decision to Treat Study Group**
Dr. Z Adhya: Kings, London
Dr. H Alachkar.: Salford
Dr. D Arnold: Sheffield
Dr. C Chopra.: Edinburgh
Dr. N Conlon.: St James’s, Dublin
Dr. T Coulter.: Belfast
Dr. J Darroch.: Liverpool
Dr. S Deacock.: Guildford
Dr. L Devlin.: Belfast
Dr. E Drewe.: Nottingham
Dr. M Duddridge: Leicester
Dr. W Egner.: Sheffield
Dr. S Goddard: Manchester
Dr. P Gordins: Hull
Dr. S Grigoriadou.: Barts, London
Prof. B Grimbacher.: Royal Free, London
Dr. G Hayman: Epsom
Dr. R Herriot.: Aberdeen
Dr. R Jain.: Oxford
Dr. S Johnston: Bristol
Dr. Y Karim: Guildford
Dr. P Kelleher.: Brompton, London
Dr. M Keogan.: Beaumont, Dublin
Dr. H Longhurst: Barts, London
Dr. S Noorani: West Birmingham
Dr. S Patel.: Oxford
Dr. R Sargur: Sheffield
Prof. S Seneviratne: Royal Free, London
Dr. C Sewell.: Lincoln
Dr. A Shrimpton: Sheffield
Dr. G Spickett.: Newcastle
Dr. M Thomas.: Glasgow
Dr. J Unsworth.: Bristol
Dr. P Vijayadurai.: Preston

## Appendix 1

### Immunoglobulin decision to treat?

Introduction.

The decision as to whether individual patients require immunoglobulin replacement therapy (IGRT) is not always easy to address. Multiple factors come into play including assessment of patient well-being, co-morbidity and laboratory testing. Guidance in this area is not absolute and UKPIN is keen to determine the views and cumulative experience of the consultant body in the UK.

The purpose of this survey is to establish what factors most influence colleagues to commence IGRT in adult patients, when strict diagnostic criteria for CVID, XLA, and other well-defined PIDs are not fulfilled. Definition of this patient group is challenging, but would include patients with slightly low total serum IgG, M or A levels, IgA or IgG subclass deficiency, or impaired responses to immunization. We are also interested in your approach to patients suspected of secondary antibody deficiency following drug therapy or haematological disorders.

The survey is neither a test of knowledge of current guidelines, nor the relative merit of different laboratory systems, but simply an attempt to delineate current practice across the UK by consultant immunologists looking after adult (> 18 year old) patients suspected of primary or secondary antibody deficiency. It is expected that there will be variations in practice across the countries, and if that is confirmed, that will be of value in informing our practice.

All data will be treated confidentially; however, it is hoped that the individual/center anonymised data will merit submission for publication; however, data submission will only occur with the consent and acknowledgment of all contributors.

Thank you in advance for your participation.

1. How often do you see patients as described above, in whom you are asked to consider immunoglobulin replacement therapy (IGRT)?
  Almost every clinic
  Quite often
  Occasionally
  Rarely
  Other (please specify)
2. Do you see referrals from other specialties (e.g. haematology, rheumatology) with suspected secondary antibody deficiency related to disease or drug therapy?
  Almost every clinic
  Quite often
  Occasionally
  Rarely
  Other (please specify)
3. In assessing immune function, prior to commencing IGRT do you undertake test immunisation?
  In all patients
  In the majority of patients
  In a minority of patients
  Rarely
  Never
  Any Comments?
4. If undertaking test immunisation, which of the following vaccines do you regularly use?
  Pneumococcal capsular polysaccharide (Pneumovax II or generic)
  Meningitis ACWY conjugated
  Prevenar 13
  Tetanus toxoid (in combined vaccine)
  Haemophilus Influenza B (HiB)
  Salmonella Typhi Vi
  Other (please specify)
5. If you use both Prevenar 13 and Pneumovax II. In which order do you administer these?
  Pneumovax II first then Prevenar 13
  Prevenar 13 first then Pneumovax II
  No fixed order
  Administer both simultaneously
  Other (please specify)
6. How long after immunisation do you recommend “post vaccination samples” be taken?
  2 weeks
  4 weeks
  8 weeks
  no fixed interval
  Other (please specify)
7. In assessment of pneumococcal antibody responses, which methodology do you use?
  Total IgG anti-pneumococcal antibody
  Total IgG2 anti pneumococcal antibody
  Pneumococcal serotype specific antibodies (ELISA)
  Pneumococcal serotype specific antibodies (bead based assay)
  Other (please specify)
8. In interpretation of pneumococcal serotype specific antibody (PSSA) results, which “cut-off” levels do you use?
  0.35 mcg/ml
  0.5 mcg/ml
  1.3 mcg/ml
  A combination of the above
  Other (please specify)
9. What percentage of serotypes do you expect to exceed your chosen “cut-off” in a normal response (choose the %threshold closest to your current practice)?
10. How do you regard the value of pneumococcal antibody response testing in the decision to commence IGRT?
  I regard this as a definitive diagnostic test
  I rely on this in the majority of cases
  I rely on this to support my clinical assessment
  I rely on this, unless it appears to conflict with the clinical history
  I am sceptical of the value and disregard this in some cases
  I quite often disregard this
  Other (please specify)
11. How do you rank the following factors in influencing your decision to commence IGRT (1=most important, 14 = least important)?
  Number of self-reported chest/sinus/ear infections
  Number of courses of antibiotics (patient reported)
  Number of courses of antibiotics (GP or pharmacy confirmed)
  Number of hospital admissions with infection
  Number of positive sputum cultures
  Types of bacteria isolated in sputum
  Radiologically proven pneumonia
  Presence of bronchiectasis
  Other structural lung disease
  Results of Immunisation studies
  Self-reported “well-being”
  Formal quality of life assessment (including validated questionairs, visual analogue scales etc)
  Total serum IgG level
  Failure to improve on antibiotic prophylaxis
12. In assessing patients for possible immunoglobulin replacement therapy, do you undertake a high resolution CT scan of chest?
  In all cases at initial assessment
  In the majority of cases at initial assessment
  Only in those cases where a daily productive cough suggests the presence of bronchiectasis
  Only in those cases where immunological investigation in inconclusive.
  Rarely.
  Other (please specify)
13. Prior to commencing IGRT in this group, do you prescribe prophylactic antibiotics?
  In all patients
  In the majority of patients
  In a minority of patients
  Rarely
  Never
  Other (please specify)
14. How would you rank the following antibiotics as suitable for prophylaxis in this patient group (1= most suitable)?
  amoxicillin
  azithromycin
  ciprofloxacin
  co-amoxiclav
  cefalexin
  co-trimoxazole
  doxycyclin
  penicillin
15. If a patient fails to improve on a trial of prophylactic antibiotics, do you consider a trial of IGRT?
  In all patients
  In the majority of patients
  In a minority of patients
  rarely
  never
16. What initial dose of IGRT do you use?
17. After what time interval would you usually assess response to immunoglobulin replacement therapy to determine whether it should be continued?
  3 months
  6 months
  9 months
  12 months
  18 months
  24 months
  Other (please specify)
18. How do you rank these factors in assessing a successful response to IGRT (1 = most important, 7 = least important)?
  Trough/random serum IgG levels
  Reduction in frequency of reported infections
  Reduction in antibiotic prescription frequency
  Reduction in hospital admissions
  Improvement in self-reported well being
  Formal quality of life assessment (standardised questionnaire, visual analogue scale etc)
  Other
19. Please provide the following details about yourself
  Name
  Centre
  Year of Consultant Appointment
  Any additional comments you would like to make?
